# A Comprehensive Eye-Tracking System Toward Large FOV HMD

**DOI:** 10.3390/s26051402

**Published:** 2026-02-24

**Authors:** Jiafu Lv, Di Zhang, Ke Han, Qi Wu, Sanxing Cao

**Affiliations:** 1 State Key Laboratory of Media Convergence and Communication, Communication University of China, Beijing 100024, China; 2 School of Data Science and Media Intelligence, Communication University of China, Beijing 100024, China; 3 Optics Department, IMT Atlantique, 29238 Brest, France

**Keywords:** eye tracking, near-eye sensing, wide field of view (wide FOV), gaze estimation, virtual reality

## Abstract

Eye tracking in virtual reality (VR) head-mounted displays poses substantial engineering challenges, particularly under immersive display configurations with large fields of view (FOV), where optical layout, illumination, and image acquisition impose nontrivial system constraints. To address these design constraints, we present an integrated near-eye eye-tracking prototype tailored for immersive VR headsets, combining customized hardware components and a real-time software pipeline. The proposed system integrates optimized near-eye illumination and image acquisition with a pupil detection module and a deep learning-based gaze-vector estimation model, forming a real-time software pipeline for stable end-to-end gaze mapping under fixed calibration conditions. Under identical system settings, calibration procedures, and gaze-point mapping conditions, we evaluate the proposed gaze-vector estimation model through a controlled model-level ablation. The attention-enhanced model achieves an average angular deviation of 1.15°, corresponding to a 61.4% relative reduction compared with a baseline ResNet-152 model without attention. To demonstrate the usability of the system outputs at the application level, we further implement a real-time visualization example that integrates pupil diameter, gaze vectors, and blink events to depict the temporal evolution of eye-movement signals. This work provides a cost-effective and reproducible engineering reference for near-eye eye-movement acquisition and visualization in immersive VR settings and serves as a technical foundation for subsequent interaction design or behavioral analysis studies.

## 1. Introduction

Eye tracking is a core sensing capability [1,2] in virtual reality (VR) systems and is widely used in applications such as gaze-contingent rendering [3,4], interaction input [5,6], user-state analysis [7,8], and visual cognition research [9]. Most existing studies and system implementations have been developed for head-mounted displays with moderate fields of view [2], under which conventional near-eye eye-tracking solutions can already achieve relatively stable performance. With the increasing demand for immersive experiences, some VR headsets have begun to adopt optical designs that significantly expand the field of view in recent years, with their coverage approaching the limits of human natural vision [10]. Such display configurations do not introduce fundamentally new perceptual tasks; rather, they place stronger demands on the engineering constraints associated with near-eye imaging. These constraints include increased off-axis viewing angles, stronger peripheral distortions, and more pronounced non-uniformity in infrared illumination [11]. Under such wide field-of-view display configurations, commonly used pupil center–corneal reflection (PCCR) methods, standard camera placements, and conventional infrared illumination schemes impose stricter requirements on the consistency of eye-image quality and the stability of gaze-mapping performance [12]. Consequently, it becomes necessary to re-examine near-eye eye-tracking implementations from a system design perspective under wide-display field-of-view conditions and to explore feasible approaches for supporting stable, real-time eye-movement sensing within this engineering setting.

Despite continuous progress in eye-tracking technologies in recent years, the design and validation of most existing systems have largely been conducted under moderate field-of-view near-eye imaging conditions, and their applicability to more extreme display configurations remains insufficiently explored. Prior work typically assumes relatively stable imaging geometry and illumination conditions; however, when the display field of view is substantially expanded, maintaining these conditions places increasing demands on system-level implementation [13]. Under wide-display field-of-view optical structures, near-eye imaging becomes more susceptible to factors such as increased off-axis viewing angles [12], stronger reflection interference [14], and variations in pupil appearance [15], thereby imposing higher requirements on pupil-boundary extraction and feature stability. While some existing prototype systems or commercial solutions can operate under such conditions, their performance characteristics are often tightly coupled to specific optical layouts or usage scenarios, which limits their flexibility when applied to diverse wide-FOV display configurations [16,17]. In addition, mainstream integrated eye-tracking modules typically adopt fixed designs in terms of camera placement, illumination geometry, and algorithmic parameters, which limits their adaptability to non-standard display configurations or research-oriented systems. Although some high-end headsets incorporate built-in eye-tracking capabilities, their closed-system architectures and higher costs make them more suitable as end products rather than as experimental platforms for investigating eye-movement sensing characteristics under varying display configurations. Based on these considerations, it is necessary to examine the feasibility of constructing near-eye eye-tracking solutions under wide-display field-of-view conditions from a system implementation perspective, and to analyze the practical constraints and design trade-offs encountered in this engineering setting.

Against this background, this paper presents a near-eye eye-tracking system designed for wide-display field-of-view configurations from a system implementation perspective, under wide field-of-view display constraints, with coordinated design of both the hardware imaging scheme and the algorithmic processing pipeline. The goal of this work is not to introduce new eye-tracking task definitions but to examine the feasibility of supporting real-time eye-movement sensing under engineering conditions associated with substantially expanded display fields of view. On the hardware side, the system adopts a monocular near-eye imaging module combined with a ring-shaped infrared illumination layout to enhance the edge visibility of the pupil region under complex imaging conditions. The camera position inside the head-mounted display is adjusted according to structural and optical constraints, enabling the acquisition of eye images with consistent quality across common ranges of ocular rotation. On the algorithmic side, we implement an eye-tracking pipeline consisting of three modules: a pupil detection method based on image morphology and geometric constraints, a deep learning model for gaze direction regression, and a gaze-point mapping method based on multi-point calibration. This process is used to mitigate the impact of imaging variations on eye-movement feature extraction and estimation within the adopted system configuration. Experimental results show that, under the adopted system configuration and experimental setup, the system operates stably at 120 FPS and achieves an average gaze-vector error of approximately 1.15°, while maintaining consistent gaze-point mapping accuracy. In addition, an application example is implemented based on the system outputs to illustrate visualization and analysis of eye-movement information in immersive tasks. This example is intended to demonstrate the usability of the system outputs at the application level, rather than to provide a quantitative evaluation of attention or cognitive states. The main contributions of this paper are summarized as follows:

1. System implementation. We develop a near-eye eye-tracking prototype system tailored for VR head-mounted displays with wide-display fields of view. The hardware design incorporates targeted adjustments to camera placement and infrared illumination layout to support high-frame-rate eye-image acquisition under wide-FOV constraints.
2. Algorithmic pipeline. We implement an eye-tracking algorithmic pipeline adapted to near-eye imaging characteristics, including pupil detection, gaze-vector estimation, and gaze-point mapping, and evaluate its performance under the same system configuration.
3. Engineering validation. Under the selected display configuration, we validate the feasibility of real-time gaze-vector estimation and gaze-point mapping under fixed calibration, providing a reusable system implementation for eye-movement sensing research in wide field-of-view display settings.

## 2. Related Work

### 2.1. Near-Eye Eye Tracking in VR

Near-eye eye tracking in virtual reality typically falls into two categories distinguished by the field of view of the headset. Conventional FOV systems, generally around 90°–110°, represent the majority of existing commercial and research platforms [18]. Recent developments in wide-FOV headsets extend the field of view beyond 120°, introducing different optical and geometric conditions. The following subsections review representative work in both settings to outline the technical work and system characteristics relevant to near-eye eye tracking.

#### 2.1.1. Eye Tracking in Conventional-FOV VR

Most near-eye eye-tracking systems have been developed for conventional-FOV VR headsets, whose field of view typically ranges between 90° and 110° [19]. Under these optical conditions, the user’s eye remains relatively close to the central region of the lens, and both the illuminated area and the effective imaging range are geometrically constrained [20]. These characteristics shape the hardware designs widely adopted in current systems: inward-facing infrared cameras are commonly positioned near the lens housing to maintain a consistent view of the eye [21,22], infrared LEDs are used to provide stable illumination and enhance pupil visibility, and the enclosed headset shell helps reduce ambient interference and reflections.

Software pipelines in this setting generally operate on the assumption that eye images exhibit stable appearance characteristics. Pupil contours remain well defined, illumination patterns vary minimally across the usable range of eye rotation, and gaze directions fall within a relatively narrow angular span. Consequently, existing methods—spanning pupil detection, geometric or learning-based gaze-vector estimation, and regression-driven gaze-point mapping—achieve high reliability and low computational overhead. These properties make conventional-FOV pipelines suitable for a wide variety of VR applications, while also establishing algorithmic expectations that are increasingly challenged as the field-of-view expands [23].

#### 2.1.2. Eye Tracking in Wide-FOV VR

Wide-FOV VR headsets expand the field of view beyond roughly 120° and frequently employ large Fresnel or hybrid lenses to achieve immersion [24]. These optics reshape the near-eye imaging geometry: the eye is observed under more oblique viewing angles [25], the pupil becomes increasingly elliptical toward the periphery, and illumination varies strongly across the expanded eyebox [26]. Reflections caused by Fresnel segments or tilted viewing rays also appear more frequently, making raw eye images less uniform and harder to interpret.

To cope with these changes, prior research has examined several hardware adaptations. Some systems reposition cameras deeper inside the lens housing [27] or at steeper viewing angles to preserve visibility at large eye rotations [28]. Others adopt wide-angle sensors or multi-camera configurations [29] to increase coverage. Illumination modules have also been modified by distributing LEDs around the lens rim [30] or adding baffles to reduce Fresnel-induced glare [31]. Although these designs improve coverage and contrast, they typically introduce new trade-offs. The cameras positioned off-axis yield stronger perspective distortion, wide-angle lenses reduce effective resolution, and expanded illumination layouts increase sensitivity to assembly tolerances or reflections from housing structures.

Software approaches have attempted to compensate for these optical variations. Prior work has explored deformable pupil models that handle elongated shapes at oblique angles [32], learning-based gaze-vector estimation trained with wide-angle datasets [33,34], and locally adaptive calibration functions that treat central and peripheral regions differently [35]. These techniques mitigate some of the variability but still face systematic limitations. Pupil contours may become too distorted for reliable segmentation, corneal reflections often drop out entirely at extreme angles, and mapping functions become increasingly nonlinear and difficult to generalize across users or headsets. As a result, tracking accuracy and stability consistently decline in the periphery despite algorithmic enhancements.

Together, these observations show that wide-FOV eye tracking presents both hardware layout and software algorithm challenges that conventional designs cannot fully resolve. These challenges and previous work inspired our subsequent design decisions regarding optical configuration and eye-tracking algorithm flow.

### 2.2. Gaze Estimation Algorithms

Existing near-eye gaze estimation pipelines typically consist of pupil detection, gaze-vector estimation, and gaze-point mapping. Prior work has developed well-established techniques for these stages under conventional-FOV imaging conditions, but their assumptions become increasingly unstable when applied to wide-FOV VR.

Pupil detection methods commonly rely on dark-pupil cues, elliptical contour fitting, or learned segmentation models, all of which assume relatively uniform illumination and near-central viewpoints [36]. In wide-FOV settings, however, the pupil often becomes highly deformed at oblique angles [37,38], eyelashes and eyelids introduce frequent partial occlusions, and reflections from large Fresnel lenses reduce local contrast. These issues undermine threshold-based and contour-based detectors and lead to instability in appearance-based approaches. To address these limitations, our system employs a multi-stage pupil detection strategy with filtering and compensation steps designed to maintain reliability under asymmetric illumination and off-axis deformation.

Gaze-vector estimation traditionally relies on geometric eye models or PCCR-based formulations, while recent work has incorporated deep regression networks [39,40,41]. Although these methods perform well when the eye-camera geometry is close to linear and corneal reflections remain visible, their accuracy deteriorates rapidly in wide-FOV conditions where glints disappear at large gaze angles and the mapping between image features and gaze direction becomes increasingly nonlinear. To handle these effects, our system adopts a ResNet-152-based estimator enhanced with attention mechanisms to improve feature extraction under varying head poses and illumination changes.

Mapping the estimated gaze vector to display coordinates typically uses polynomial regression [42,43], SVR mappings [44], or grid-based calibration [45]. Such methods rely on smooth geometric distortion and dense calibration samples—conditions rarely satisfied in wide-FOV headsets, where peripheral regions exhibit strong nonlinearities and calibration errors accumulate. In response, our system applies a two-dimensional regression model combined with a nine-point calibration procedure, enabling accurate mapping across a broader gaze range while preserving real-time performance.

### 2.3. Eye-Tracking Applications in VR

Eye tracking plays an important role in virtual reality, serving as both an interaction modality and a foundation for performance optimization, attention modeling, and user behavior analysis. In interaction contexts, gaze has been widely used for target selection, pointing assistance, and multimodal input fusion [46,47,48], helping reduce manual workload and improve operational efficiency. In rendering systems, gaze-contingent techniques leverage precise fixation information to enhance foveal detail while dynamically lowering peripheral resolution, thereby reducing computational cost [3,49]. At the same time, numerous studies have exploited eye-movement patterns to infer attention, engagement, or cognitive load and to analyze users’ visual behavior, spatial scanning strategies, and task-related perceptual responses in dynamic VR environments.

Despite their diversity, these applications share several common data requirements: coverage across a wide gaze range, stable high-frame-rate acquisition, reliable pupil localization even at large rotation angles, and consistent gaze-point mapping over extended display regions. However, current headsets provide limited support for these needs under wide-FOV optical conditions, restricting the deployment of research prototypes and experimental paradigms that rely on high-quality gaze measurements. Thus, the present work develops a wide-FOV near-eye gaze-tracking system and demonstrates its use through an example application for visual-attention state detection. This example illustrates the system’s applicability to behavior analysis and cognition-oriented VR scenarios and highlights its potential to support a broader range of gaze-driven research tasks.

## 3. Hardware Design

Wide-FOV near-eye imaging imposes strict constraints on camera placement, illumination geometry, and internal structural layout. Large Fresnel lenses, expanded eyebox requirements, and oblique viewing angles reshape the attainable imaging volume and limit the feasible positions for sensing components. As a result, conventional eye-tracking hardware architectures designed for narrow-FOV headsets cannot be directly reused.

This chapter presents the hardware design of our wide-FOV gaze-tracking system, including its overall architecture, image acquisition module, infrared illumination module, and supporting shell structure. For each module in the system, this paper describes the underlying design motivations and the implementation constraints introduced by wide field-of-view conditions and provides reference specifications related to the system implementation to help readers understand the overall design rationale and implementation context.

### 3.1. Overall Hardware Architecture

Under wide field-of-view VR headset conditions, near-eye imaging systems are subject to significant spatial and optical constraints in the placement of cameras and infrared light sources. To achieve compact and controllable system integration under such constrained conditions, this work adopts a monocular sensing configuration as the starting point for the near-eye eye-tracking system design. This choice is motivated partly by practical engineering constraints and partly by prior empirical practices in eye-tracking research, where monocular configurations have been used for gaze estimation under limited hardware resources or near-eye imaging setups. In addition, from a physiological perspective, the high degree of coordination between the human eye during gaze behavior—such as that described by Hering’s law [50] of equal innervation—provides a reasonable basis for using signals from a single eye as an approximate representation at the level of the gaze direction. Based on these combined considerations, a monocular sensing scheme is adopted in the system design to explore its engineering feasibility and integration characteristics in wide-FOV headset scenarios.

**Figure 1:**
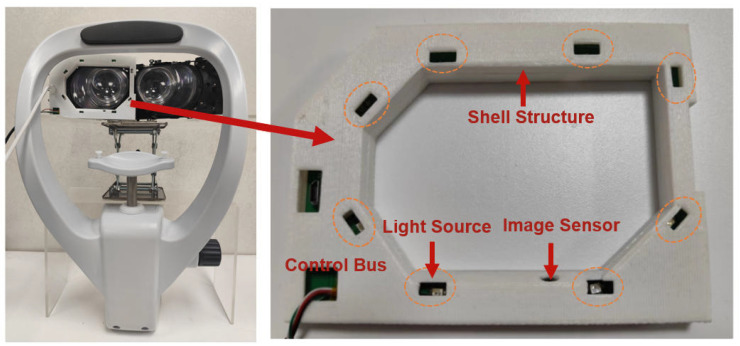
The overall hardware architecture of the wide field-of-view near-eye eye-tracking prototype.

As shown in Figure 1, our hardware design consists of three tightly integrated modules: an image-acquisition module, an infrared illumination module, and a custom shell structure that positions these components within the headset. The shell provides the mechanical framework for mounting the camera and IR emitters at predefined angles, ensuring stable imaging across large eye-rotation ranges. The shell also provides passive physical support for the participant’s head, which constrains large head movements during the experiments. The illumination module delivers directed near-infrared lighting to enhance pupil visibility, while the image-acquisition module collects high-contrast eye images for subsequent processing. The overall architecture is designed to fully consider the physical constraints imposed by the large Fresnel lenses and expanded field of vision in the wide field-of-view headset, while ensuring structural stability and consistent performance during experiments to meet experimental requirements.

### 3.2. Image Acquisition Module

Based on project requirements, this study adopts the Pimax 5K Plus wide field-of-view virtual reality head-mounted display as the system platform, on top of which the near-eye imaging module is independently designed and integrated. The headset employs large Fresnel lenses and provides an expanded-display field of view; its optical structure and limited internal space impose explicit geometric constraints on the placement of near-eye sensing components. Under wide-FOV near-eye imaging conditions, positioning the camera directly in front of or beside the eye can easily introduce significant occlusion or cause the eye to move outside the effective imaging range of the sensor. Therefore, in the design of the image acquisition module, we took into account the limitations of the internal structure and optical layout of the head-mounted display and installed the camera at the lower part of the shell of the eye-tracking device (as shown in the image sensor in the right part of Figure 1). This placement helps keep the eye near the center of the captured image during a large range of eye rotation and ensures continued visibility of the pupil and iris during significant eye movements. Compared to the side-mounted layout, the bottom-mounted configuration reduces the geometric distortion caused by off-axis imaging to a certain extent, allowing the acquired pupil contour to maintain a relatively consistent geometric shape under different gaze directions, thus providing a stable input for subsequent pupil detection and gaze-vector estimation. Based on this, the early image processing stages only require limited geometric corrections to meet the needs of subsequent processing.

When determining the camera mounting pose, the tilt angle is not arbitrarily chosen but is jointly constrained by the field-of-view characteristics of the near-eye imaging module and the internal housing structure of the headset. The selected tilt orientation must ensure that the pupil remains within the effective imaging region of the sensor across typical eye movements, while simultaneously avoiding occlusion caused by the edges of the Fresnel lens or other internal structural components. Under these constraints, the camera is installed within an engineering-feasible range of tilt angles, allowing stable and consistent image quality to be maintained within the limited available space.

The module employs a near-infrared global-shutter sensor operating at 120 FPS with 640 × 480 resolution (OV7251). The sensor’s global-shutter exposure provides stable frame timing under fast eye movements, while its low-light sensitivity and dynamic range satisfy the illumination variability inside wide-FOV headsets, where reflections and position-dependent shading are common. The paired low-distortion lens (<1% barrel distortion) preserves accurate pupil shape representation, enabling reliable pupil detection and gaze-vector estimation without heavy preprocessing.

Overall, the image acquisition module is optimized for consistent visibility of the pupil across the expanded eyebox of wide-FOV VR headsets, providing stable and high-quality inputs for the subsequent stages of the gaze-tracking pipeline.

### 3.3. Light Source Module

The illumination module is designed to provide stable near-infrared lighting across the eye region under the geometric and optical constraints of wide-FOV headsets. Because of the large Fresnel lenses and the expanded eyebox, single-direction illumination tends to produce shadows, non-uniform brightness, and strong reflections along the lens grooves. To mitigate these effects, our system adopts an array of eight near-infrared LEDs, distributed around the inner perimeter of the headset lens. This ring-like arrangement helps maintain coverage across different eye rotations and reduces localized shading caused by lens curvature or user motion.

Each LED is embedded within a custom housing structure (Figure 1), which fixes its relative mounting angle and position while primarily constraining the geometric relationship between the illumination paths and the imaging field of view. This design allows the emitted near-infrared light to stably cover the eye region without introducing occlusion to the camera’s imaging path. The infrared LEDs used in this system operate at a wavelength of 850 nm, a band that is widely adopted for illumination in camera-based near-eye eye-tracking systems. The wavelength selection took into account both imaging requirements and user safety, among other engineering factors. On one hand, 850 nm lies outside the range of human visual perception, which helps reduce interference with users’ visual experience during operation. On the other hand, under typical near-eye imaging illumination conditions, this wavelength has been extensively used in eye-tracking research and system implementations and is commonly operated under controlled power levels and working modes. Based on these imaging and engineering constraints, 850 nm infrared LEDs are selected as the illumination source for near-eye imaging. The combination of distributed placement and near-infrared illumination supports consistent pupil visibility across different gaze directions, providing more uniform inputs for subsequent pupil detection and gaze estimation stages.

### 3.4. Shell Structure Design

The shell structure is designed to securely integrate the eye-tracking components into the wide-FOV VR headset while preserving user comfort and accommodating the geometry of the optical system. The headset employs elliptical Fresnel lenses (approximately 9.3 cm × 6.3 cm), whose shape and surrounding clearances restrict the available mounting space. To address these constraints, we adopt a two-layer shell design tailored to fit the internal contour of the headset.

The upper layer provides an opening for the camera module, ensuring an unobstructed optical path for image acquisition. The lower layer conforms closely to the boundary of the Fresnel lens assembly, allowing the structure to anchor firmly without interfering with the user’s field of view. This tight integration prevents positional drift of the sensing components during head motion and maintains consistent alignment between the camera, illumination sources, and the eye.

By distributing the structural load across two layers and separating optical access from mechanical fixation, the shell improves stability while reducing direct pressure on the eye region. This design supports extended use of wide-FOV headsets, whose larger optical modules and internal components may otherwise introduce discomfort or instability when additional sensing hardware is installed.

## 4. Eye-Tracking Algorithm Design

**Figure 2:**
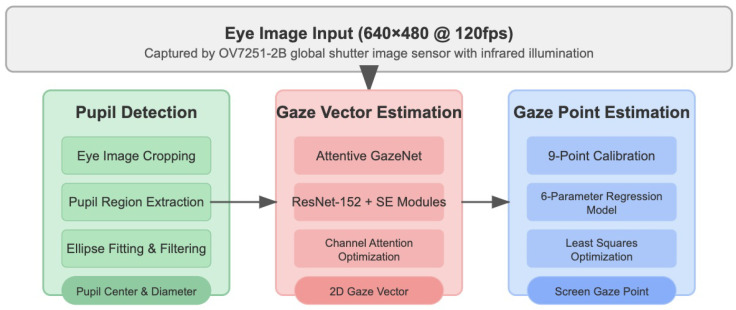
An overview of the eye-tracking process, including pupil detection, gaze-vector estimation, and gaze-point estimation.

Wide-FOV near-eye imaging introduces several challenges for eye-movement analysis, including off-axis distortion, uneven illumination, and pronounced changes in pupil appearance across gaze directions. To address these factors, our gaze-tracking pipeline is organized into three synergistic components—pupil detection, gaze-vector estimation, and gaze-point computation (Figure 2). Each component is designed with careful consideration of the geometric and photometric characteristics of wide-FOV headsets, ensuring that the extracted features remain stable under larger gaze ranges and oblique viewing angles. This section introduces the principles and implementation of the aforementioned modules and describes the system implementation adopted under the current experimental setup to support near-eye image acquisition at approximately 120 frames per second.

### 4.1. Pupil Detection

Pupil detection is a critical component of the gaze-tracking pipeline, as its output provides the basis for subsequent gaze-vector estimation and gaze-point mapping. In wide field-of-view (FOV) VR headsets, near-eye imaging conditions are complex, and the pupil region is often affected by factors such as eyelid occlusion, eyelash texture, variations in near-infrared illumination, and local reflections introduced by Fresnel lenses, which together increase the difficulty of stable pupil localization. Based on these imaging characteristics, this work adopts a pupil localization approach grounded in dark-pupil imaging, combined with contour modeling and geometric constraints.

**Figure 3:**
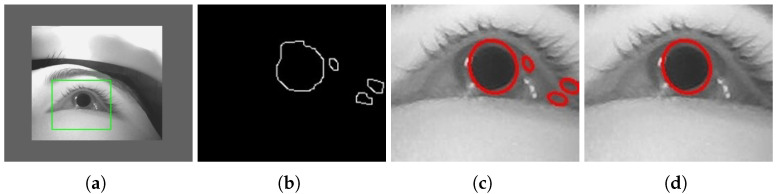
Overview of the pupil detection pipeline. (**a**) Eye region localization. (**b**) Edge detection. (**c**) Contour-based ellipse fitting. (**d**) Dual-constraint filtering using area and aspect ratio.

In the processing pipeline, boundary extraction is first performed within the cropped eye-image region by exploiting the grayscale contrast between the pupil and the surrounding iris tissue under dark-pupil imaging conditions. This step highlights intensity transitions at the pupil boundary by detecting edges formed by local grayscale variations. As illustrated in Figure 3a, this procedure is intended to extract pupil-related boundary information from complex eye images, even in the presence of eyelash occlusion, local shadows, or reflection interference, thereby providing a foundation for subsequent processing. The extracted boundary information is then organized into a set of independent contours, as shown in Figure 3b, which represent candidate pupil-boundary shapes.

Building on the extracted candidate contours, an ellipse model is applied to each contour to perform geometric fitting, leveraging the fact that the pupil region typically exhibits circular or elliptical shapes. Each fitting operation takes a set of contour points as input and returns the corresponding ellipse parameters, including the center position, the lengths of the major and minor axes, and the overall orientation. The use of an ellipse model is motivated by the observation that, under frontal viewing conditions, the pupil appears approximately circular in the image, while under off-axis eye rotations, its projection on the imaging plane gradually deforms into an elliptical shape. Based on this geometric property, ellipse fitting provides a unified parametric representation for subsequent geometric filtering and pupil localization. Example fitting results are shown in Figure 3c.

Because multiple non-pupil contours may be present simultaneously in real eye images, directly using all ellipse fitting results would introduce false detections. Therefore, after ellipse fitting, area and aspect ratio constraints are introduced into the candidate results to screen candidates that do not clearly conform to the geometric features of the pupil. First, a constraint is imposed on the projected area of each fitted ellipse to remove extremely small contours caused by noise or local texture, as well as abnormally large contours resulting from reflections or region merging. This constraint is based on the geometric prior knowledge that the pupil projection region has a finite scale in the image and filters out candidate results that are obviously too small or too large by filtering the area range. This area constraint is defined in the form of explicit upper and lower bounds to exclude candidate results with scale anomalies under the current imaging conditions; in our implementation, only contours with projected areas in the range of [700, 10,000] pixels are retained. Secondly, constraints are applied based on the aspect ratio of the ellipse to exclude candidate results whose shapes significantly deviate from the pupil projection characteristics; specifically, the ratio between the major and minor axes is constrained to the range of [0.6, 1.6] in the current system. Since the projection of the pupil onto the imaging plane is usually close to a circle, its major axis and minor axis should be at similar lengths. Therefore, this constraint is used to filter out excessively long or highly flat non-target contours. Together, the area and aspect ratio constraints further refine the ellipse fitting results, retaining only candidates that satisfy both size and shape characteristics of pupil geometry. If multiple ellipse candidates satisfy both constraints, the candidate with the largest projected area is selected as the pupil. If no candidate satisfies the constraints, the detection at the current frame is considered failed. An example of the candidate contours after filtering is shown in Figure 3d.

**Figure 4:**
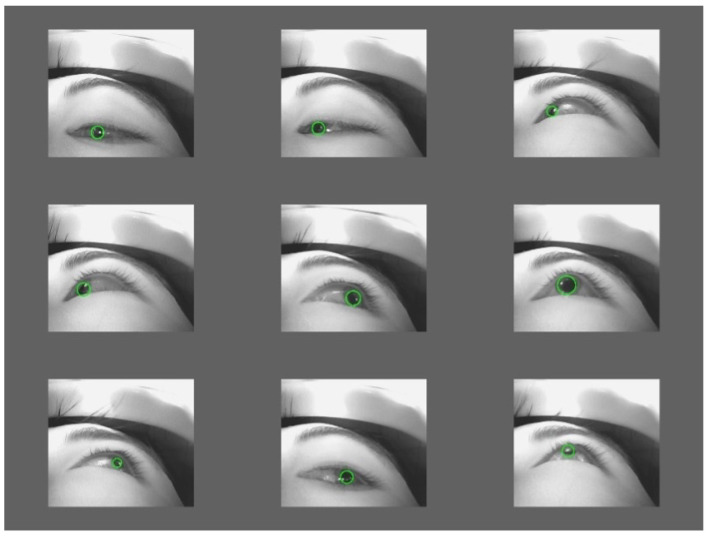
Schematic diagram of pupil detection results.

Finally, to improve the temporal continuity of the pupil detection pipeline during runtime, a simple frame-to-frame continuity-based compensation strategy is introduced at the implementation level. Specifically, the system retains the most recent valid pupil estimation from the previous frame. When the current frame fails to yield a valid pupil contour—such as during blinking or short-term occlusion—the previous estimate is temporarily reused to avoid interruptions in the output stream. This strategy is adopted as a practical engineering measure within the current system configuration and is applied during real-time operation at a frame rate of 120 fps (corresponding to an inter-frame interval of approximately 8.3 ms). Representative detection examples are shown in Figure 4 to illustrate the behavior of the pupil detection pipeline during typical runtime scenarios.

### 4.2. Gaze-Vector Estimation with Attention Mechanism

In VR eye-gaze tracking systems, gaze-vector estimation converts the visual appearance of the eye in near-eye images into the user’s gaze direction, serving as a key input for subsequent three-dimensional gaze-point computation. In wide field-of-view head-mounted displays, factors such as Fresnel lens structures, large variations in eye pose, and non-uniform infrared illumination can significantly affect near-eye imaging conditions, thereby increasing the difficulty of gaze-vector estimation. Against this background, this section employs a deep learning-based regression method to directly map eye appearance features into continuous horizontal and vertical gaze components. This supports the gaze-point estimation process under wide field-of-view display conditions and serves as the input basis for subsequent gaze-point mapping and system evaluation.

**Figure 5:**
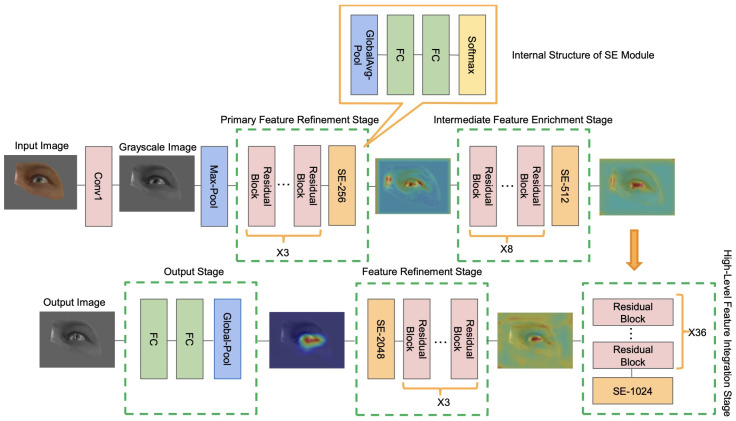
Attentive GazeNet Network architecture diagram.

We build the Attentive GazeNet on top of ResNet-152 [51] and integrate Squeeze-and-Excitation (SE) modules [52] into its main residual stages to strengthen channel-wise attention. As shown in Figure 5, ResNet-152 extracts multi-scale structural features, including pupil-edge gradients, fine-grained iris textures, and the elliptical deformations that arise under off-axis viewing. The SE blocks model inter-channel dependencies and reweight discriminative cues, enabling the network to focus on gaze-relevant regions while suppressing low-confidence areas affected by Fresnel reflections or local shadows. The attention-enhanced features are then passed to a lightweight regression head that outputs the horizontal (x) and vertical (y) gaze vectors. This architecture maintains strong representational capacity while supporting stable real-time inference. During training, input images are normalized and geometrically aligned to improve robustness across eye poses and illumination conditions.

Subsequently, the proposed gaze-vector estimation model was evaluated on a gaze direction regression task using the UnityEyes synthetic near-eye dataset. The UnityEyes dataset provides accurately annotated gaze directions for each eye image frame, enabling supervised learning of gaze-vector components. In the experiments, the dataset was split into training and validation sets using a fixed ratio for model optimization and performance assessment. To ensure a fair comparison, the proposed Attentive GazeNet and the baseline ResNet-152 model were trained under identical data partitioning strategies, training epochs, and optimization settings. In this task, the horizontal and vertical components of the gaze vector were used as regression targets to represent the user’s gaze direction under near-eye imaging conditions. Since gaze-vector estimation is a continuous regression problem, mean squared error (MSE) was adopted as both the loss function and the evaluation metric, as defined in Equation (1), to measure the regression error between the predicted gaze-vector components and the ground-truth annotations.

(1) $${\mathrm{Loss}}_{\mathrm{MSE}}=\frac{1}{N}\sum_{i=1}^{N}{\left(y_{i}-y_{i}^{\prime }\right)}^{2}$$

where *N* denotes the total number of training samples, $y_{i}$ represents the ground-truth value, and $y_{i}^{\prime }$ denotes the predicted value produced by the network. Notably, this error is defined in the normalized gaze-vector component space rather than in angular units or screen-space coordinates.

Experimental results show that the proposed Attentive GazeNet achieves a lower regression error on this task, with an MSE of 0.0071, compared to 0.0421 for the baseline model, corresponding to an error reduction of approximately 83%. This result indicates that incorporating attention mechanisms helps strengthen the representation of gaze-related features, thereby reducing regression error in gaze-vector estimation.

### 4.3. Gaze-Point Estimation

After obtaining the gaze vector, the system further computes the user’s actual point of regard on the virtual display plane. Although the proposed system is designed for virtual reality head-mounted display applications, gaze-point estimation and accuracy evaluation in this study are conducted on a predefined two-dimensional target plane, where target positions are specified in the plane coordinate system and remain fixed throughout the experiments. Given the fixed position of the near-eye camera, this problem can be formulated as establishing a mapping on a 2D plane from the pupil center (represented through the gaze vector) to the display coordinates. Considering prior work and the real-time performance requirements of the system, we adopt a lightweight and well-established second-order polynomial regression model for the mapping. This approach provides strong engineering stability under fixed near-eye optical configurations, maintaining accuracy while avoiding the computational cost associated with multi-camera geometric modeling or high-order polynomial fitting.

The mapping model uses a six-parameter quadratic polynomial that relates the pupil-center coordinates to the corresponding gaze point on the screen, as shown in Equation (2). This representation captures the nonlinear relationships introduced by off-axis imaging while keeping the model complexity under control.

(2) $$\left\{\begin{array}{c} x_{g}=a_{0}+a_{1}x_{p}+a_{2}y_{p}+a_{3}x_{p}^{2}+a_{4}x_{p}y_{p}+a_{5}y_{p}^{2}\\ y_{g}=b_{0}+b_{1}x_{p}+b_{2}y_{p}+b_{3}x_{p}^{2}+b_{4}x_{p}y_{p}+b_{5}y_{p}^{2} \end{array}\right.$$

where $\left(x_{p},y_{p}\right)$ represent pupil-centered gaze-vector coordinates and $\left(x_{g},y_{g}\right)$ represent target plane gaze points. In this study, the gaze reference is not obtained via an external eye-tracking device but is defined using a task-based ground truth. During the experiment, participants are instructed to fixate on predefined visual targets; accordingly, the target location on the display plane is used as a reference point for gaze-to-target mapping at that moment.

To determine the twelve polynomial coefficients (six for each axis), a nine-point calibration procedure is employed. Each calibration point is displayed using a cross-shaped fixation marker, which effectively guides users’ visual focus toward the precise center. During calibration, participants sequentially fixate on predefined locations, and the system records the associated gaze vectors, forming the equation set shown in Equation (3).

(3) $$\left\{\begin{array}{c} RA=X_{g}\\ RB=Y_{g} \end{array}\right.$$

where *R* represents the conversion matrix, *A* and *B* are the coefficient vectors, and $X_{g}/Y_{g}$ are the calibration point coordinates. These paired observations are then used to solve for the polynomial coefficients using a least-squares approach.

**Figure 6:**
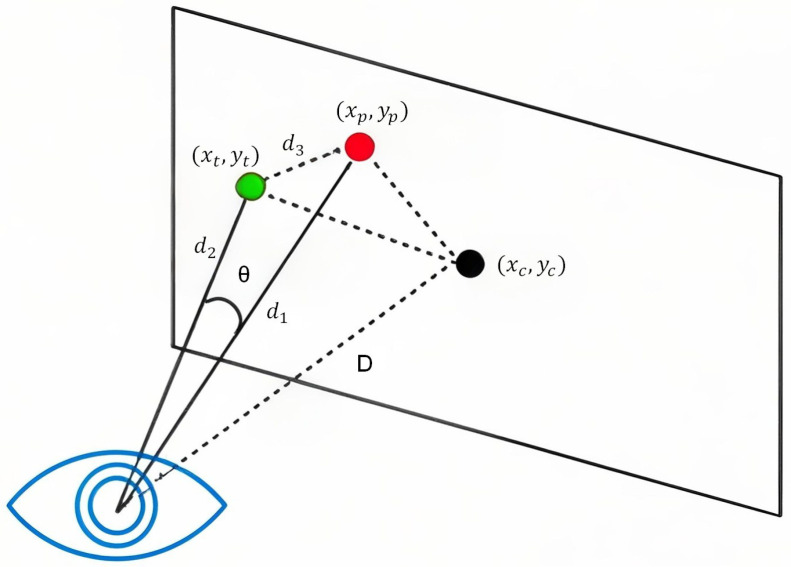
Geometric principle of angular error computation for gaze-point estimation.

To evaluate the performance of gaze-point estimation, we use the angular deviation between the estimated and actual gaze points as the accuracy metric. Specifically, as illustrated in Figure 6, all visual targets in the experiment are rendered on a predefined virtual target plane within the head-mounted display (HMD) coordinate system. During the experiment, participants were instructed to fixate sequentially on the targets while their head position was passively supported by the experimental rig, which constrained large head movements during the task. Under this experimental setting, the target position on the display plane is therefore treated as the ground-truth reference for gaze-point mapping at the corresponding moment. The specific calculation procedure is summarized in Equation (4):

(4) $$\left\{\begin{array}{cc} d_{1} & =\sqrt{{\left(x_{p}-x_{c}\right)}^{2}+{\left(y_{p}-y_{c}\right)}^{2}+D^{2}},\\ d_{2} & =\sqrt{{\left(x_{t}-x_{c}\right)}^{2}+{\left(y_{t}-y_{c}\right)}^{2}+D^{2}},\\ d_{3} & =\sqrt{{\left(x_{p}-x_{t}\right)}^{2}+{\left(y_{p}-y_{t}\right)}^{2}},\\ \theta & =\arccos\left(\frac{d_{1}^{2}+d_{2}^{2}-d_{3}^{2}}{2d_{1}d_{2}}\right). \end{array}\right.$$

where $\left(x_{t},y_{t}\right)$ represent the participant’s actual gaze coordinates, and $\left(x_{p},y_{p}\right)$ represent the system-predicted gaze coordinates. The eye position is approximated by its projection onto the target plane, denoted as $\left(x_{c},y_{c}\right)$, and the distance *D* between the eye and the target plane is known and fixed in the experiment. Based on this geometric configuration, the spatial distances between the eye and the target gaze point as well as the estimated gaze point are first computed. The angular deviation θ between the estimated gaze direction and the target gaze direction is then derived using the law of cosines, converting the planar gaze-point discrepancy into a corresponding visual angle.

Then, twenty participants (10 male, 10 female; mean age = 23.5 years, SD = 3.04), all without known ocular or health conditions that could affect the results, were recruited from a local university. The experiment consisted of two phases: calibration and accuracy assessment. During the gaze-point estimation experiment, participants were instructed to fixate on the targets while their head position was passively supported by the experimental rig, providing partial physical constraint against large head motions. No explicit head pose compensation was applied; therefore, the reported angular errors primarily reflect the performance of gaze-point estimation under near-head-fixed conditions. In the calibration phase, participants fixated on a sequence of displayed markers to establish the mapping parameters. The calibration procedure was performed only once at the beginning of the experiment, and the resulting mapping parameters were kept fixed throughout the subsequent accuracy evaluation stage. No recalibration was performed during testing. Once calibration was completed, the system automatically entered the accuracy testing phase, where participants sequentially fixated on nine predefined target positions. During the experimental test, the coverage of the gaze target on the screen was ±36.8°. The nine target locations used during the accuracy evaluation stage were identical to those used in the calibration stage. This design aims to assess the stability and repeatability of the gaze-point mapping model under fixed calibration conditions, rather than its generalization performance to unseen target locations. For each position, the system recorded ten samples of gaze-point data, followed by outlier removal to enhance measurement reliability. Outliers were removed using a statistical threshold-based method. For each target location, a gaze-point sample was considered an outlier and discarded if its deviation from the mean of all samples at that location exceeded two standard deviations. The processed results were then visualized, and the angular error between predicted and ground-truth points was computed.

**Figure 7:**
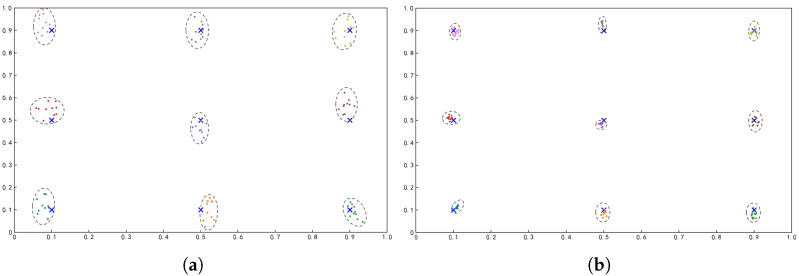
Comparison of subject-specific gaze-point estimation results based on two models, where the elliptical contour represents the covariance ellipse (BCEA) result for the corresponding target.The horizontal and vertical axes represent the mean deviation (in pixels) in the corresponding directions. (**a**) Gaze-point estimation results based on ResNet-152. (**b**) Gaze-point estimation results based on Attentive GazeNet.

Based on statistics from twenty participants, the two models produced mean angular errors of 2.98° and 1.15° under identical mapping and calibration conditions, indicating a reduction of approximately 61.4%. This demonstrates that Attentive GazeNet significantly improves overall gaze-point accuracy compared to ResNet-152. Furthermore, as illustrated in Figure 7, the predicted gaze points from ResNet-152 show a more dispersed distribution around the nine targets, whereas Attentive GazeNet produces notably more concentrated and accurate predictions. Combining the BCEA fitting results, it can be observed that the spatial distribution of gaze-point estimation errors shows a clear pattern related to target location: the estimation error is smallest at the central target, followed by targets located along the horizontal and vertical axes, while the four corner points exhibit the greatest spatial dispersion.

### 4.4. Comparison with Representative Near-Eye Gaze-Tracking Systems

This section provides a system-level comparative analysis of several representative near-eye gaze-tracking approaches, with the goal of clarifying the design trade-offs among different systems in terms of sensing hardware configuration, output information type, and applicable display field-of-view (FOV) ranges. Table 1 summarizes key system characteristics reported in prior work, including average gaze angular error, sensing hardware configuration, types of eye-movement information provided by the system, and the display field of view (display FOV). It should be noted that some prior methods do not explicitly report the display FOV in their original publications, or instead describe coverage ranges that are not directly associated with a specific display configuration. To avoid conceptual ambiguity, the display FOV is listed in Table 1 only when it is explicitly reported in the corresponding literature; otherwise, the entry is left unspecified.

Regarding the selection of comparison methods, this work considers two representative system design paradigms as references. The first category includes event-camera-based gaze estimation systems, which typically achieve high estimation accuracy but rely on specialized sensing hardware and do not provide pupil-related outputs. The second category consists of systems based on conventional imaging sensors combined with multi-illumination configurations. These approaches follow more mature hardware pathways, but their sensing range and output capabilities are constrained by specific optical geometries and design objectives. These two categories reflect different trade-offs between hardware complexity and sensing capability in current near-eye gaze-tracking systems. Within this design space, the system in this paper supports both gaze-direction estimation and pupil detection output in its system design and is implemented for virtual reality applications under large-display field-of-view conditions.

## 5. Application

**Figure 8:**
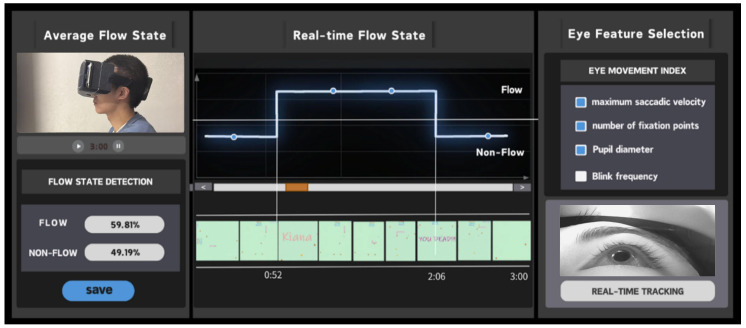
Real-time visual attention detection interface showing attention state waveforms and tracking metrics.

In this section, we developed a real-time visualization interface to present the system’s eye-tracking output and its temporal variation characteristics during immersive tasks, with the goal of improving the observability and interpretability of the overall tracking process. The interface visualizes multiple eye-movement-related signals produced by the system to illustrate changes in users’ gaze behavior over time. The term “flow state” is used solely as an example label for interface demonstration, referring to gaze patterns associated with relatively high levels of visual engagement [55], rather than a quantitative detection or inference of users’ psychological states. As shown in Figure 8, the interface integrates the outputs of the three core modules—pupil detection, gaze-vector estimation, and gaze-point computation—into a unified visualization framework. It comprises three main views: an overview of gaze behavior across the entire viewing session, a real-time visualization of gaze behavior over time, and an interactive panel for selecting and displaying different eye-movement features. Through this interface, the system’s responses to natural eye behaviors such as saccades, smooth pursuit, sustained fixation, and short-term occlusion can be directly observed.

During the operation of this visualization example, the system’s stable performance under high-refresh-rate conditions can be observed, including smooth interface updates, continuous output of effective pupil localization results within a wide gaze angle range, and response to instantaneous eye-movement events such as blinking. These observations reflect the overall stability of the proposed near-eye tracking pipeline under real-time operating conditions. It should be emphasized that this demonstration is based on an application-level runtime example and is intended to illustrate the usability and extensibility of the system outputs in immersive VR scenarios, rather than to provide quantitative evaluation or validation of blink detection, attention states, or related behavioral measures. The example primarily serves to support the exploration of gaze-driven interaction prototypes, behavior analysis workflows, and system integration for eye-tracking-related applications.

## 6. Discussion

The findings of this study suggest that, under the adopted system configuration, constructing a near-eye eye-tracking system for ultra-wide-FOV displays involves simultaneously addressing the complex couplings introduced by optical structure, illumination distribution, and image characteristics. Unlike conventional head-mounted displays with ≤110° FOV, wide-FOV optical systems introduce stronger peripheral distortions and segmented reflections, which can lead to substantial spatial variations in pupil-boundary quality and gaze-related image features, particularly at larger gaze angles. Within the evaluated setup, our hardware and algorithmic design indicates that coordinated adjustment of the camera mounting angle, IR-illumination geometry, and feature-modeling strategies can mitigate these coupled effects and contribute to more consistent system behavior under the selected wide-FOV configuration.

From a mechanistic perspective, the observed performance of the pupil detection module under the evaluated wide-FOV imaging conditions appears to be associated with its integrated “edge enhancement→contour filtering→ellipse fitting” architecture. Compared with approaches that rely primarily on thresholds or single-feature cues, this structure consolidates fragmented edges into coherent geometric shapes, enabling more reliable pupil characterization in the presence of uneven illumination, eyelash occlusions, or localized reflections. Similarly, the attention-enhanced deep regression model exhibits improved performance in gaze-vector prediction under the adopted experimental conditions: the SE module reweights channel features such that the model can suppress the influence of lens reflections, bright spots, and low-contrast regions, thereby learning gaze-direction patterns that are more consistent within the evaluated data distribution. Together, these two components provide the gaze-mapping stage with low-noise, structurally consistent inputs, allowing the six-parameter regression model to maintain effective fitting capability within the geometry of the evaluated wide-FOV system.

Notably, the results also reveal several synergistic relationships between hardware and algorithms within the current system configuration. For example, the eight-point IR-illumination configuration not only increases pupil-image contrast but also improves the separability of edge features learned by the deep model under the adopted imaging conditions. Likewise, the downward camera layout expands the visible eye region and contributes to more consistent coordinate relationships during gaze-point mapping in the evaluated setup. These observations suggest that the observed performance gains of the proposed system do not arise from any single module in isolation, but instead emerge from the overall consistency achieved through joint optimization of hardware and software components within this specific engineering configuration.

It should be emphasized that the present study is positioned as an engineering feasibility investigation conducted under a fixed-system configuration and calibration procedure. The reported performance metrics primarily reflect target-referenced gaze-mapping repeatability within the evaluated setup, rather than generalized point-of-regard accuracy across varying gaze dynamics or alternative system configurations. In this work, the ultra-wide field of view is treated as a system design context that motivates the proposed hardware–software integration, rather than as a validated performance regime or an experimentally analyzed independent variable. Accordingly, the findings are intended to inform system-level design trade-offs and implementation feasibility rather than to establish FOV-dependent performance laws or behavioral generalizations.

## 7. Limitations and Future Work

First, our work adopts a pupil detection pipeline based on geometric constraints to support stable real-time operation under wide field-of-view near-eye imaging conditions. The geometric constraints used in this study (e.g., area and aspect-ratio constraints) are empirically determined based on the current camera resolution, optical layout, and illumination configuration, with the goal of ensuring robust performance under the specific system setup. This design choice may limit the generalizability of the results, as these parameters may need to be adjusted when the imaging resolution, optical structure, or camera placement differs. Future work will further examine the relationship between parameter selection and imaging conditions and explore more adaptive parameter modeling or learning-based pupil detection strategies.

Second, to maintain output continuity under high frame-rate operation, a simple frame-to-frame continuity-based compensation strategy is introduced at the implementation level. This design choice primarily serves to support stable real-time operation during experiments and does not include a quantitative analysis of error propagation under conditions such as frequent blinking, rapid saccades, or prolonged occlusion. Future studies will model the temporal characteristics of detection failures in greater detail and evaluate their impact on downstream gaze-point estimation accuracy.

Third, the experimental evaluation in this study is based on a relatively homogeneous participant group composed primarily of young university students. While this setup supports reliable within-subject evaluation of system feasibility and stability under wide-FOV display conditions, it may limit the generalizability of the findings to broader user populations. Future work will include more diverse participant groups and consider factors such as age-related ocular differences, the use of corrective lenses, and individual visual conditions to further investigate how user variability interacts with system design choices.

In this study, gaze-point accuracy is primarily evaluated using average angular error as an end-to-end system-level metric. While this measure effectively reflects overall gaze-tracking performance under wide-FOV VR conditions, it does not fully characterize the spatial structure of estimation errors or explicitly separate systematic and random error components. Although we provide an initial spatial characterization based on target-specific distributions and BCEA fitting (Figure 7), future research will incorporate finer-grained spatial analyses and error decomposition to better understand performance boundaries and variability under wide-FOV conditions.

Finally, our work focuses on system integration and real-time feasibility at the level of a research prototype evaluated under controlled experimental conditions. The reported 120 FPS refers to the near-eye image acquisition rate, which is sufficient to support stable real-time operation during the experiments. However, deployment-level performance metrics such as end-to-end system latency and power consumption are not explicitly quantified. These metrics depend strongly on specific hardware platforms and integration configurations. Future work will conduct systematic evaluations of latency and energy consumption under application- or product-oriented system setups.

## 8. Conclusions

This work presents a near-eye eye-tracking system designed for wide field-of-view (FOV) VR headsets, combining a tailored infrared illumination layout with a coordinated hardware–algorithm pipeline for pupil detection and gaze estimation. By jointly considering optical constraints, sensor placement, and algorithmic processing, the system achieves stable pupil capture and consistent gaze-point estimation under wide-FOV imaging conditions. The results suggest that, under wide-FOV near-eye configurations, reliable eye-tracking performance depends more on maintaining structural consistency across the hardware–algorithm interface than on increasing model complexity alone. From a system implementation perspective, careful coordination between imaging geometry, illumination design, and estimation models plays a critical role in sustaining stable real-time operation. In addition, an application-level visualization example is implemented to illustrate how the system outputs can be integrated and explored in immersive VR scenarios. Rather than serving as a validated behavioral or attention inference method, this example demonstrates the usability and extensibility of the proposed system as a technical foundation for gaze-driven interaction prototyping and eye-movement analysis. Overall, this work provides a practical system implementation and engineering reference for near-eye eye-tracking research under wide-FOV VR display configurations. It should be noted that the reported results reflect engineering feasibility and target-referenced gaze-mapping repeatability under a fixed system configuration, rather than generalized gaze accuracy across varying eye dynamics or display field-of-view conditions.

## Figures and Tables

**Table 1:** System-level comparison with representative near-eye gaze-tracking approaches.

Method	Accuracy (°)	Hardware Dependency	Output Type	Display FOV (°)
DVS-NN [53]	0.91	Event-based Camera	Gaze	96° × 64°
3D Model [54]	1.23	Three Light + RGB Camera	Gaze	-
This work	1.15	Eight Light + NIR Eye-tracking Camera (OV7251, global shutter)	Gaze + pupil	200° × 85°

## Data Availability

The data presented in this study are not publicly available due to operational restrictions but are available from the corresponding author upon reasonable request.
